# Current potential health benefits of sulforaphane

**DOI:** 10.17179/excli2016-485

**Published:** 2016-10-13

**Authors:** Jae Kwang Kim, Sang Un Park

**Affiliations:** 1 Division of Life Sciences, College of Life Sciences and Bioengineering, Incheon National University, Incheon, 406-772, Korea; 2 Department of Crop Science, Chungnam National University, 99 Daehak-ro, Yuseong-gu, Daejeon, 305-764, Korea

## ⁯

Dear Editor,

Sulforaphane [SFN: 1-isothiocyanato-4-(methylsulfinyl)butane] belongs to the isothiocyanate class of phytochemicals. Glucoraphanin, a glucosinolate precursor of SFN, is a glucosinolate found in cruciferous vegetables such as broccoli, cabbage, cauliflower, and kale. All glucosinolates are composed of a basic structure consisting of a β-D-thioglucose group, a sulfonated oxime group, and an amino acid-derived side chain. Glucosinolates are activated by enzyme-dependent hydrolysis to their respective isothiocyanates. SFN (molecular formula C_6_H_11_NOS_2_) is the biologically active isothiocyanate produced by the metabolism of glucoraphanin by the enzyme myrosinase (Fahey et al., 2015[11]). 

SFN is one of the most frequently studied plant-derived isothiocyanate organosulfur compounds. It has been reported to exhibit a wide range of biological effects including antioxidant (Fahey and Talalay, 1999[10]), antimicrobial (Johansson et al., 2008[19]), anticancer (Amjad et al., 2015[4]), anti-inflammatory (Greaney et al., 2016[14]), anti-aging (Sikdar et al., 2016[45]), neuroprotective (Tarozzi et al., 2013[47]), and antidiabetic (Lee et al., 2012[26]). 

SFN shows a range of biological activities and health benefits in humans, has been found to be a very promising chemopreventive agent against not only a variety of cancers such as breast, prostate, colon, skin, lung, stomach, and bladder but also against cardiovascular and neurodegenerative diseases and diabetes (Yang et al., 2016[53]). In this present study, we reviewed the most recent studies on the biological and pharmacological activities of SFN (Table 1(Tab. 1)) (References in Table 1: Pal and Konkimalla, 2016[34]; Zhao et al., 2016[55]; Wu et al., 2016[52]; Sasaki et al., 2016[39]; Jiang et al., 2016[17]; Hernández-Rabaza et al., 2016[15]; Sikdar et al., 2016[45]; Li et al., 2016[28]; Thaler et al., 2016[48]; Lan et al., 2016[23]; Shehatou and Suddek, 2016[42]; Townsend and Johnson, 2016[49]; Qi et al., 2016[37]; Abbas et al., 2016[1]; Kikuchi et al., 2015[21]; Atwell et al., 2015[6]; Ma et al., 2015[30]; Kim et al., 2015[22]; Wang et al., 2015[50]; Lubecka-Pietruszewska et al., 2015[29]; Brown et al., 2015[7]; Carrasco-Pozo et al., 2015[8]; Ambrecht et al., 2015[3]; Lavich et al., 2015[24]; Waston et al., 2015[51]; Shirai et al., 2015[43]; Prasad and Mishra, 2015[36]; Li et al., 2015[27]; Cipolla et al., 2015[9]; Angeloni et al., 2015[5]; Oguz et al., 2015[33]; Noh et al., 2015[32]; Shang et al., 2015[41]; Horwacik et al., 2015[16]; Shokeir et al., 2015[44]; Alzoubi et al., 2015[2]; Gabriel et al., 2015[13]; Kee et al., 2015[20]; Rizzo et al., 2014[38]; Pan et al., 2014[35]; Sayed et al., 2014[40]; Singh et al., 2014[46]; Maeda et al., 2014[31]; Zhang et al., 2014[54]; Lee et al., 2014[25]; Fimognari et al., 2014[12]; Jo et al., 2014[18]).

| Key findings | Reference |
|---|---|
| The immunomodulatory effects of SFN clearly indicate that it alleviates chronic inflammatory diseases by targeting monocytes/macrophages. | Pal and Konkimalla, 2016 |
| SFN attenuates experimental contrast-induced nephropathy in vitro and in vivo. This effect is suggested to be mediated by activation of the nuclear factor erythroid-derived 2-like 2 (Nrf2) antioxidant defence pathway. | Zhao et al., 2016 |

EXCLI Journal 2016;15:571-577 – ISSN 1611-2156
Received: June 28, 2016, accepted: August 24, 2016, published: October 13, 2016

| Key findings | Reference |
|---|---|
| SFN inhibited hepatocellular carcinoma cell proliferation in a dose- and time-dependent manner. All our findings indicate that SFN is a promising and safe strategy for treating hepatocellular carcinoma. | Wu et al., 2016 |
| SFN treatment increases the liver 3α-hydroxysteroid dehydrogenases, accelerates the degradation of blood dihydrotestosterone (DHT), and subsequently blocks the suppression of hair growth by DHT. | Sasaki et al., 2016 |
| The chemopreventive effect of SFN is associated with its inhibition of histone deacetylase (HDAC) activity, which attenuates lung cancer growth. These findings suggest that SFN may be a promising therapeutic agent for lung cancer by the inhibition of HDAC. | Jiang et al., 2016 |
| Treatment with SFN could be useful for improving cognitive function in patients with cirrhosis with minimal or clinical hepatic encephalopathy. | Hernández-Rabaza et al., 2016 |
| The use of SFN as a protective agent against ultraviolet damage is a novel application, and it appears to be a very promising emerging ingredient in anti-aging drugs and cosmetics. | Sikdar et al., 2016 |
| SFN protects cardiomyocytes from hypoxia/reoxygenation injury in vitro, most likely by activating the silent information regulator 1 (Sir1) pathway and subsequently inhibiting endoplasmic reticulum (ER) stress-dependent apoptosis. | Li et al., 2016 |
| SFN epigenetically stimulates osteoblast activity and diminishes osteoclast bone resorption, thereby shifting the bone homeostasis balance to favor bone acquisition, mitigation of bone resorption, or both in vivo. Thus, SFN is a member of a new class of epigenetic compounds that could be considered novel strategies to counteract osteoporosis. | Thaler et al., 2016 |
| SFN remarkably suppressed cell growth and enhanced cell death in chemo-resistant xenografts in the nude mouse model. Collectively, the present study suggests that the clinical efficacy of temozolomide-based chemotherapy of temozolomide-resistant glioblastoma may be improved by combination therapy with SFN. | Lan et al., 2016 |
| SFN ameliorates the progression of high cholesterol diet-induced atherosclerotic lesions and vascular dysfunction, possibly via its lipid-lowering and antioxidant effects and suppression of nuclear factor-kappa B (NF-κB)-mediated inflammation. | Shehatou and Suddek, 2016 |
| SFN is a beneficial supplement that may be useful for reducing microglial-mediated neuroinflammation and the oxidative stress associated with aging. | Townsend and Johnson, 2016 |
| SFN exerts protective effects against lipopolysaccharide-induced acute lung injury through the nuclear factor-erythroid 2-related factor 2 (NFE2L2)/antioxidant response element (ARE) pathway. Thus, SFN may be a potential candidate for use in the treatment of acute lung injury. | Qi et al., 2016 |
| The SFN-mediated modification of chromatin composition and structure associated with target gene expression provides a new mechanism by which dietary phytochemicals may exert their chemopreventive activity. | Abbas et al., 2016 |
| Dietary supplementation with broccoli sprout extract containing the SFN precursor, glucoraphanin, is likely to be highly effective in improving liver function through the reduction of oxidative stress. | Kikuchi et al., 2015 |
| Glucoraphanin supplementation for a few weeks is safe but may not be sufficient to produce changes in breast tissue tumor biomarkers. Future studies using larger sample sizes should evaluate alternative dosage regimens to improve dietary SFN strategies for breast cancer chemoprevention. | Atwell et al., 2015 |
| SFN suppresses the inflammatory response by inhibiting the NF-κB signaling pathway in a rat model of focal cerebral ischemia and, therefore, may be a potential therapeutic agent for the treatment of cerebral ischemia injury. | Ma et al., 2015 |

EXCLI Journal 2016;15:571-577 – ISSN 1611-2156
Received: June 28, 2016, accepted: August 24, 2016, published: October 13, 2016

| Key findings | Reference |
|---|---|
| SFN may inhibit human colon cancer progression and cancer cell angiogenesis by inhibiting hypoxia inducible factor-1α (HIF-1α) and vascular endothelial growth factor (VEGF) expression. Taken together, these results indicate that SFN is a new and potent chemopreventive drug candidate for treating patients with human colon cancer. | Kim et al., 2015 |
| SFN attenuated the cytotoxicity of cadmium selenide (CdSe) quantum dots (QDs) in both human hepatocytes and the mouse liver, and this protection was associated with the induction of the Nrf2 pathway and autophagy. | Wang et al., 2015 |
| SFN activates DNA methylation-silenced tumor suppressor genes in breast cancer cells. Therefore, SFN may be a supportive adjuvant therapy with the anti-cancer drug, clofarabine and, therefore, might increase its effectiveness in solid tumor treatment. | Lubecka-Pietruszewska et al., 2015 |
| The potential usefulness of the blockade of bronchoconstrictor hyperresponsiveness in some types of asthmatics by phytochemicals such as SFN has been reported. | Brown et al., 2015 |
| The antioxidant effects of SFN in mouse plasma and hippocampal formations is evidenced by the increased catalase and superoxide dismutase activity, as well as the increased adenosine triphosphate (ATP) production by hippocampal mitochondria. Furthermore, these effects likely underlie SFN's anticonvulsant mechanisms of action. | Carrasco-Pozo et al., 2015 |
| In vehicle-treated mice, ischemia/reperfusion (I/R) injury produced a marked thinning of the inner retinal layers, which, however, appeared to be significantly reduced following SFN treatment. Therefore, SFN may be beneficial in the treatment of retinal disorders associated with I/R injury. | Ambrecht et al., 2015 |
| SFN reversed the iron-induced decrease in the mitochondrial fission protein, DNM1L, as well as hippocampal synaptophysin levels, leading to a recovery of the associated recognition memory impairment. These findings suggest that SFN should be further investigated as a potential agent for the treatment of cognitive deficits associated with neurodegenerative disorders. | Lavich et al., 2015 |
| SFN does not directly stimulate autophagy or cell death in metastatic prostate cancer cells under physiologically relevant conditions. However, it supports the involvement of important in vivo effectors that mediate its prostate cancer suppression. | Waston et al., 2015 |
| SFN may have prophylactic and therapeutic effects on cognitive impairment in schizophrenia. Therefore, the dietary intake of SFN-rich broccoli sprouts during the juvenile and adolescent stage may prevent the onset of adult psychosis. | Shirai et al., 2015 |
| SFN was also found to efficiently scavenge hydrogen peroxide by converting it into water. Thus, the mechanism of action of SFN as an excellent antioxidant has been revealed. | Prasad and Mishra, 2015 |
| SFN protected the vascular endothelial cells against lysophosphatidylcholine-induced injury by enhancing the antioxidative capabilities mediated by Nrf-2 translocation. | Li et al., 2015 |
| Daily administration of free SFN shows promise in managing biochemical recurrences of prostate cancer after radical prostatectomy. | Cipolla et al., 2015 |
| SFN demonstrates pleiotropic behavior, owing to its effects on different cellular targets, suggesting a potential role in preventing or counteracting multifactorial neurodegenerative disorders such as Alzheimer's disease (AD). | Angeloni et al., 2015 |
| SFN reduced the liver oxidative stress induced by I/R injury. Furthermore, histological injury of the liver was reduced by SFN administration. However, SFN showed no significant effects on the remote organ injuries induced by IR. | Oguz et al., 2015 |
| SFN plays a protective role against acetaminophen-mediated hepatotoxicity through antioxidant effects mediated by heme oxygenase-1 (HO-1) induction. SFN has preventive actions against oxidative stress-mediated liver injury. | Noh et al., 2015 |

| Key findings | Reference |
|---|---|
| SFN ameliorated experimental diabetic nephropathy, at least in part, via the glycogen synthase kinase 3-beta (GSK3β)/Fyn (tyrosine kinase)/Nrf2 signaling pathway. | Shang et al., 2015 |
| Combined treatment of cells with SFN and 3-methyladenine (3-MA) proved to be effective in decreasing cell viability, through a mechanism that may involve early SFN-induced autophagy, followed by induction of apoptosis and inhibition of autophagy by 3-MA. | Horwacik et al., 2015 |
| SFN showed more potent renoprotection against I/R injury than ischemic preconditioning (Ipre) did, and this effect might involve synergism between them at the molecular but not functional level. | Shokeir et al., 2015 |
| SFN stimulates suicidal erythrocyte death or eryptosis, which may, at least partially, be due to the stimulation of $Ca^{(2+)}$ entry and ceramide formation. | Alzoubi et al., 2015 |
| SFN stimulates proteasome activity and autophagy in normal and Hutchinson-Gilford progeria syndrome (HGPS) fibroblast cultures. Specifically, SFN enhances progerin clearance by autophagy and reverses the phenotypic changes that are the hallmarks of HGPS. Therefore, SFN is a promising therapeutic strategy for children with HGPS. | Gabriel et al., 2015 |
| SFN has the potential to prevent cardiac hypertrophy by downregulating the transcription factors GATA-binding factor 4/6 (GATA4/6) and mitogen-activated protein kinase (MAPK) signaling pathways. | Kee et al., 2015 |
| Both SFN and klotho, a protein with multiple pleiotropic effects associated with anti-aging, enhance the antioxidant defenses, which may protect against vascular smooth muscle cell dysfunction in age-related cardiovascular diseases. | Rizzo et al., 2014 |
| SFN showed protective effects against retinal I/R, which could be attributed, at least in part, to the activation of the Nrf2/HO-1 antioxidant pathway. | Pan et al., 2014 |
| SFN alleviates D-galactosamine/lipopolysaccharide-induced liver injury, possibly by exerting antioxidant, anti-inflammatory, and antiapoptotic effects and modulating certain antioxidant defense enzymes. | Sayed et al., 2014 |
| Dietary SFN is recognized to have low toxicity and was identified for its ability to reverse abnormalities associated with autism spectrum disorder. The abnormalities included oxidative stress and lowered antioxidant capacity, depressed glutathione synthesis, reduced mitochondrial function and oxidative phosphorylation, increased lipid peroxidation, and neuroinflammation. | Singh et al., 2014 |
| Blockade of the advanced glycation end product (AGE)-receptor for AGE (RAGE) axis in pericytes by SFN might be a novel therapeutic target for the treatment of diabetic retinopathy. | Maeda et al., 2014 |
| SFN ameliorates neurobehavioral deficits by reducing cholinergic neuron loss in the brains of AD-like mice, and the underlying mechanism may be associated with neurogenesis and aluminum load reduction. These findings suggest that the phytochemical, SFN has potential usefulness in AD therapy. | Zhang et al., 2014 |
| SFN-enhanced autophagy flux provided protection against prion-mediated neurotoxicity, which was regulated by adenosine monophosphate (AMP)-activated protein kinase (AMPK) signaling pathways in human neuronal cells. This data also suggests that SFN has potential value as a therapeutic tool in neurodegenerative disorders including prion diseases. | Lee et al., 2014 |
| SFN might set the stage for the development of a novel therapeutic principle that complements the growing armature against malignancies, which would encourage the exploration of its efficacy in a broader population of patients with leukemia. | Fimognari et al., 2014 |
| SFN has antitumor effects against bladder cancer cells mediated through a reactive oxygen species (ROS)-mediated intrinsic apoptotic pathway, which suggest that ER stress and Nrf2 may represent strategic targets for SFN-induced apoptosis. | Jo et al., 2014 |

***Acknowledgements***

***Conflict of interest***

The authors declare no conflict of interest

## Acknowledgements

This work was supported by Korea Institute of Planning and Evaluation for Technology in Food, Agriculture, Forestry and Fisheries (IPET) through Agri-Bio Industry Technology Development Program, funded by Ministry of Agriculture, Food and Rural Affairs (MAFRA) (316006-5).

## Conflict of interest

The authors declare no conflict of interest

## Figures and Tables

**Table 1:**
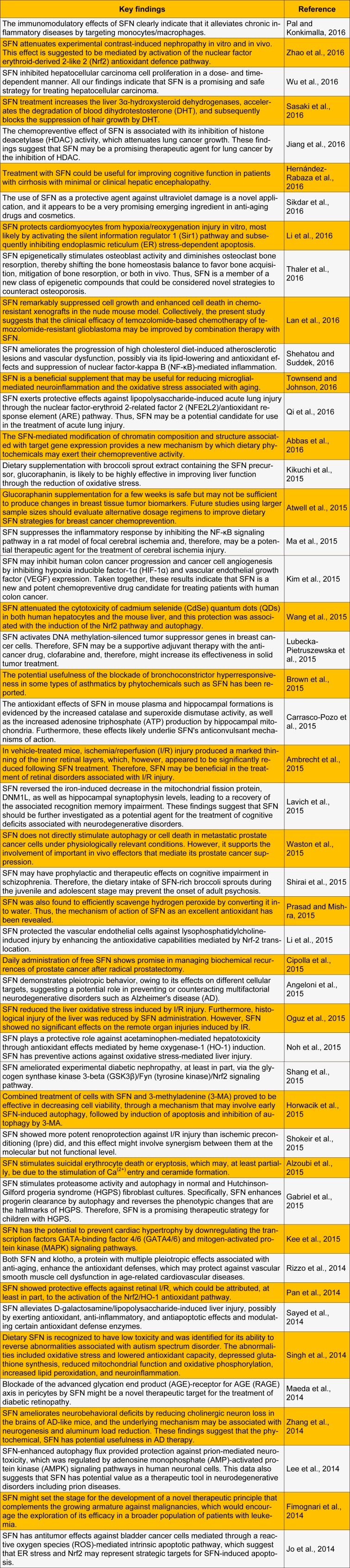
Recent studies on biological and pharmacological activities of sulforaphane (SFN)
